# Sarcopenia in glioblastoma: the imaging we need and what it tells us

**DOI:** 10.1007/s00066-024-02267-x

**Published:** 2024-08-02

**Authors:** Fabian M. Troschel, Hans Theodor Eich

**Affiliations:** Klinik für Strahlentherapie – Radioonkologie, Universitätsklinikum Münster, Albert-Schweitzer-Campus 1, 48149 Münster, Germany

Dear editor,

We thank Abdusalam and Kara for their insightful discussion [1] of our findings [2] and appreciate the opportunity to offer our thoughts.

Abdusalam and Kara discuss our determination of sarcopenia based on planning computed tomography (CT). In doing so, we follow in a tradition of high-ranking investigations that have relied upon imaging to determine the presence of sarcopenia (including publications in *Lancet Oncology, Radiology*, and *JAMA*) [2–9]. In the last 4 years alone, close to 40 investigations have used the term “CT-defined sarcopenia” according to a search on PubMed, including investigations in the *Journal of Cachexia, Sarcopenia, and Muscle*, one of the most relevant journals in the field [10]. Hence, our study’s imaging-based definition of sarcopenia is well in line with a large body of literature. We are aware that an imaging-based definition, while commonly used, is not universally applied: An increasing number of studies use definitions that rely on a combination of imaging and physical function [11, 12] or on physical function alone [13, 14]. The need for a consensus definition remains pressing, but is unmet at this time [15].

There is similar heterogeneity when it comes to the type of imaging for muscle mass assessment, though even some ultrasound-based investigations [16, 17] as well as most consensus papers [7, 11, 12] refer to computed tomography (CT) or magnetic resonance imaging (MRI) as the gold standard. The undeniable advantage of our study setup is the use of available imaging as cranial CT scans are routinely acquired for radiation planning in every glioblastoma patient. Hence, our assessment can be performed *without additional examinations* for patients. We observed a strong correlation between cranial parameters and standardized L3-level lean muscle measures, indicating the value of cranial muscle quantifications [18]. Conversely, a recent meta-analysis found low-to-moderate test accuracy for ultrasound-based sarcopenia assessment [19].

In sum, we appreciate the thoughts presented by Abdusalam and Kara. Homogenizing the definition of sarcopenia is an important inspirational goal that we share, and physical functioning tests may play important roles in this setting. However, CT-based sarcopenia definitions remain well-supported in the literature at this time, indicating the validity of our approach. In addition, despite the suggestion for ultrasound assessment, we continue to advocate the use of cranial CT imaging for body composition analysis given the availability of imaging as part of clinical routine. Nevertheless, whatever the optimal imaging modality, we appreciate the authors’ contribution to a better understanding of sarcopenia as an important risk factor in cancer patients.
